# Supplementary material for: Neonatal brain dynamic functional connectivity in term and preterm infants and its association with early childhood neurodevelopment
Source: Nat Commun. 2024 Feb 8;15:16. doi: 10.1038/s41467-023-44050-z (PMC10853532; doi:10.1038/s41467-023-44050-z)
Supplement: Supplementary file 1 — Supplementary Information [file 41467_2023_44050_MOESM1_ESM.pdf]

Supplementary Information: Neonatal brain dynamic functional connectivity in term and preterm infants and its association with early childhood neurodevelopment

Lucas G. S. França[1,2,3], Judit Ciarrusta[1,2], Oliver Gale-Grant[1,2], Sunniva Fenn-Moltu[1,2], Sean Fitzgibbon[4], Andrew Chew[2], Shona Falconer[2], Ralica Dimitrova[1,2], Lucilio Cordero-Grande[2,5,6], Anthony N Price[2], Emer Hughes[2], Jonathan O'Muircheartaigh[1,2,7], Eugene Duff[4,8,9], Jetro J Tuulari[10,11,12], Gustavo Deco[13,14,15,16], Serena J Counsell[2], Joseph V Hajnal[2], Chiara Nosarti[2,17], Tomoki Arichi[2,7,18,19], A David Edwards[2,7†], Grainne McAlonan[1†], Dafnis Batalle[1,2*†]

1) Department of Forensic and Neurodevelopmental Science, Institute of Psychiatry, Psychology & Neuroscience, King's College London, London SE5 8AF, United Kingdom

2) Centre for the Developing Brain, School of Biomedical Engineering & Imaging Sciences, King's College London, London SE1 7EH, United Kingdom

3) Department of Computer and Information Sciences, Faculty of Engineering and Environment, Northumbria University, Newcastle upon Tyne, NE1 8ST, United Kingdom

4) Oxford Centre for Functional Magnetic Resonance Imaging of the Brain, Wellcome Centre for Integrative Neuroimaging, University of Oxford, Oxford OX3 9DU, United Kingdom

5) Biomedical Image Technologies, ETSI Telecomunicación, Universidad Politécnica de Madrid, 28040 Madrid, Spain

6) Centro de Investigación Biomédica en Red de Bioingeniería, Biomateriales y Nanomedicina, Instituto de Salud Carlos III, 28029 Madrid, Spain

7) MRC Centre for Neurodevelopmental Disorders, King's College London, London SE1 1UL, United Kingdom

8) Department of Brain Sciences, Imperial College London, London W12 0BZ, United Kingdom

9) UK Dementia Research Institute at Imperial College London, London W12 0BZ, United Kingdom

10) FinnBrain Birth Cohort Study, Turku Brain and Mind Center, Institute of Clinical Medicine, University of Turku, 20500 Turku, Finland

11) Turku Collegium for Science and Medicine and Technology, University of Turku, 20500 Turku, Finland

12) Department of Psychiatry, University of Turku and Turku University Hospital, 20500 Turku, Finland

13) Center for Brain and Cognition, Computational Neuroscience Group, Department of Information and Communication Technologies, Pompeu Fabra University, 08002, Barcelona, Spain

14) Catalan Institution for Research and Advanced Studies, 08010 Barcelona, Spain

15) Department of Neuropsychology, Max Planck Institute for Human Cognitive and Brain Sciences, 04103 Leipzig, Germany

16) School of Psychological Sciences, Monash University, Melbourne VIC 3010, Australia

17) Department of Child and Adolescent Psychiatry, Institute of Psychiatry, Psychology & Neuroscience, King's College London, London SE5 8AF, United Kingdom

18) Department of Paediatric Neurosciences, Evelina London Children's Hospital, Guy's and St Thomas' NHS Foundation Trust, London SE1 7EH, United Kingdom

19) Department of Bioengineering, Imperial College London, London SW7 2AZ, United Kingdom

Supplementary Tables

Supplementary Table S1 | Association of global dynamic features (synchrony and metastability) with PMA, PND at scan, and preterm birth, using the M-CRIB atlas.

	Term (*n* = 324)				Term vs Preterm (*n* = 390)			
	PMA at scan		PND at scan		Term (*n* = 324)	Preterm (*n* = 66)	Cohen's *D*	*p*-value††
	t†	p-value†	t†	p-value†	[mean (S.D.)]			
Mean synchronisation	-0.173	0.862	1.370	0.172	0.53 (0.08)	0.48 (0.08)	0.628	*p* < 0.001*
Metastability	0.753	0.448	-2.330	0.019*	0.20 (0.02)	0.19 (0.02)	0.480	*p* < 0.001*

†GLM1 (including 324 term-born babies): $y \sim \beta_0 + \beta_1$PMA + β_2PND + β_3Sex + β_4Motion outliers (FD). ††GLM2 (including 324 term-born and 66 preterm-born babies): $y \sim \beta_0 + \beta_1$Preterm-born + β_2PMA + β_3Sex + β_4Motion outliers (FD). p-values obtained with a two-sided permutation test. * Indicates results surviving FDR multiple comparison correction with α error at 5%). PMA: Postmenstrual age. PND: Postnatal days.

Supplementary Figures

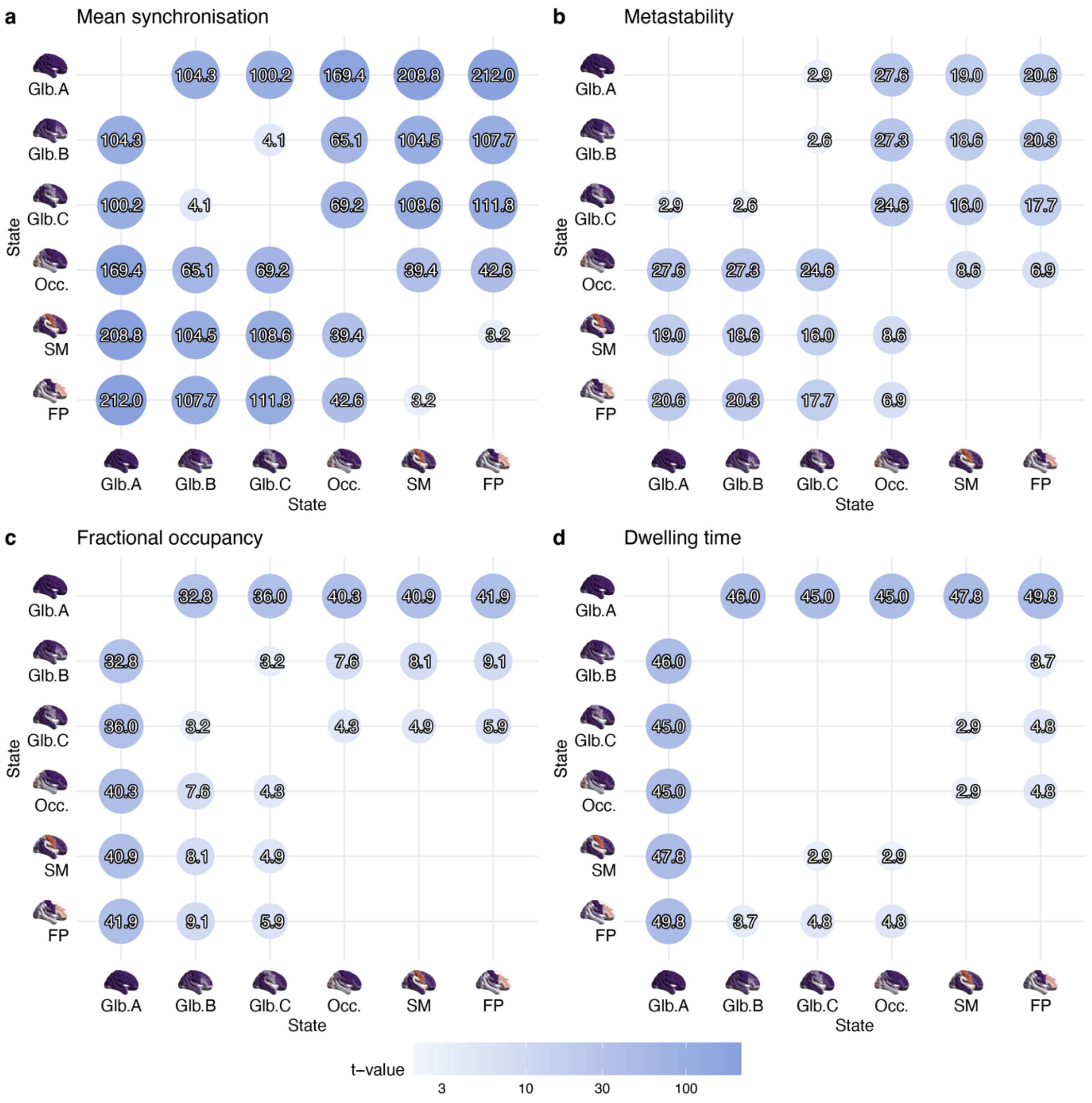

Supplementary Figure S1 | ANOVA two-sided post-hoc tests (n = 324) for **(a)** Mean synchronisation, **(b)** Metastability, **(c)** fractional occupancy, and **(d)** dwell times. Glb.: Global. Occ.: Occipital. SM: Sensorimotor. FP: Frontoparietal.

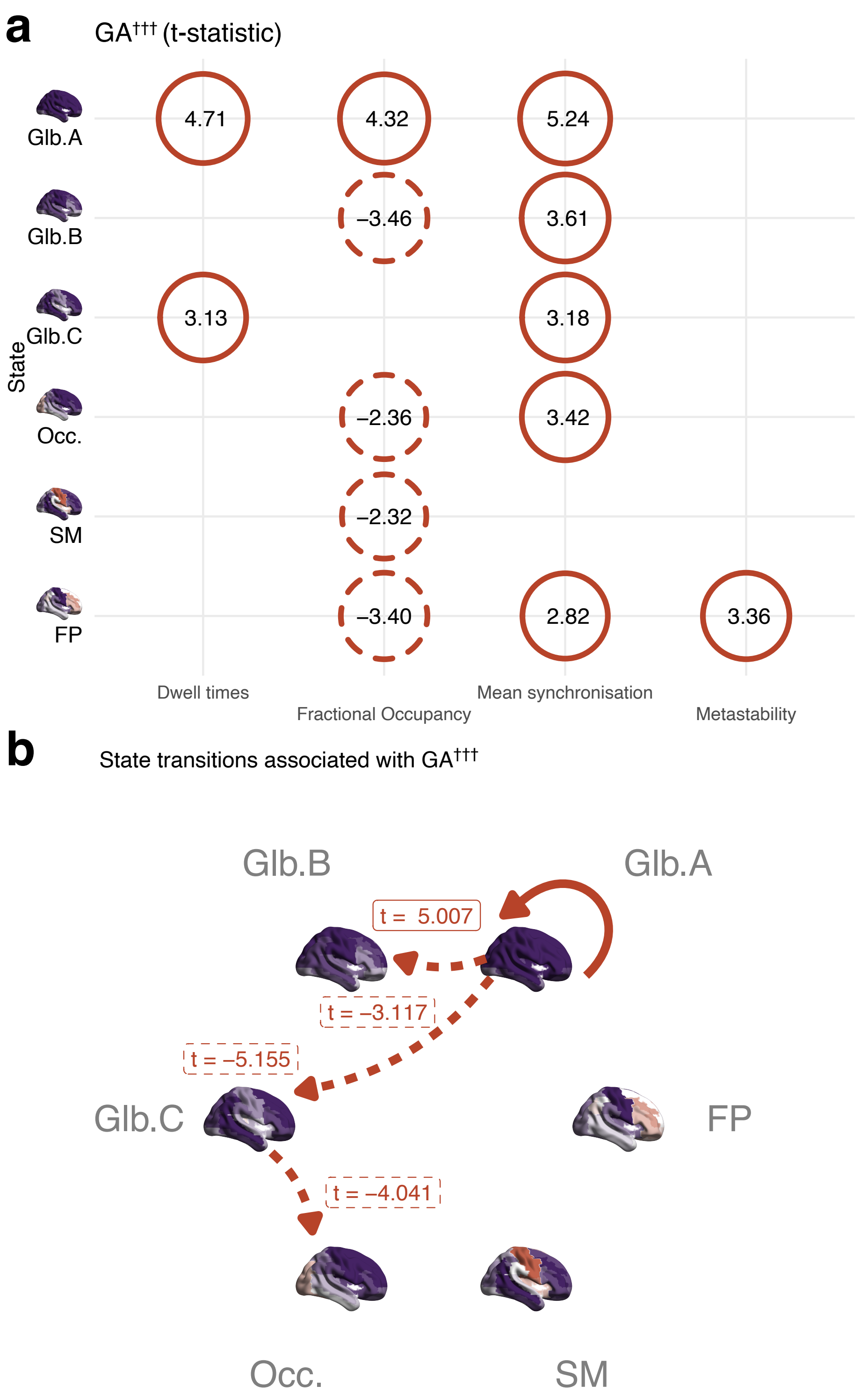

Supplementary Figure S2 | Association of GA at birth with modular features of neonatal brain dynamics (n = 390). (**a**) Summary of associations between each of the four metrics (dwell times, fractional occupancy, mean synchronisation, metastability) and GA at birth. (**b**) Association of state transitions probabilities and GA at birth. †††GLM3 (including 324 term-born and 66 preterm-born babies): $y \sim \beta_0 + \beta_1\text{GA} + \beta_2\text{PMA} + \beta_3\text{Sex} + \beta_4\text{Motion outliers (FD)}$. * $p < 0.05$. ** $p < 0.01$. *** $p < 0.001$. Glb.: Global. Occ.: Occipital. SM: Sensorimotor. FP: Frontoparietal. PMA: Postmenstrual age. PND: Postnatal days. GA: Gestational age.

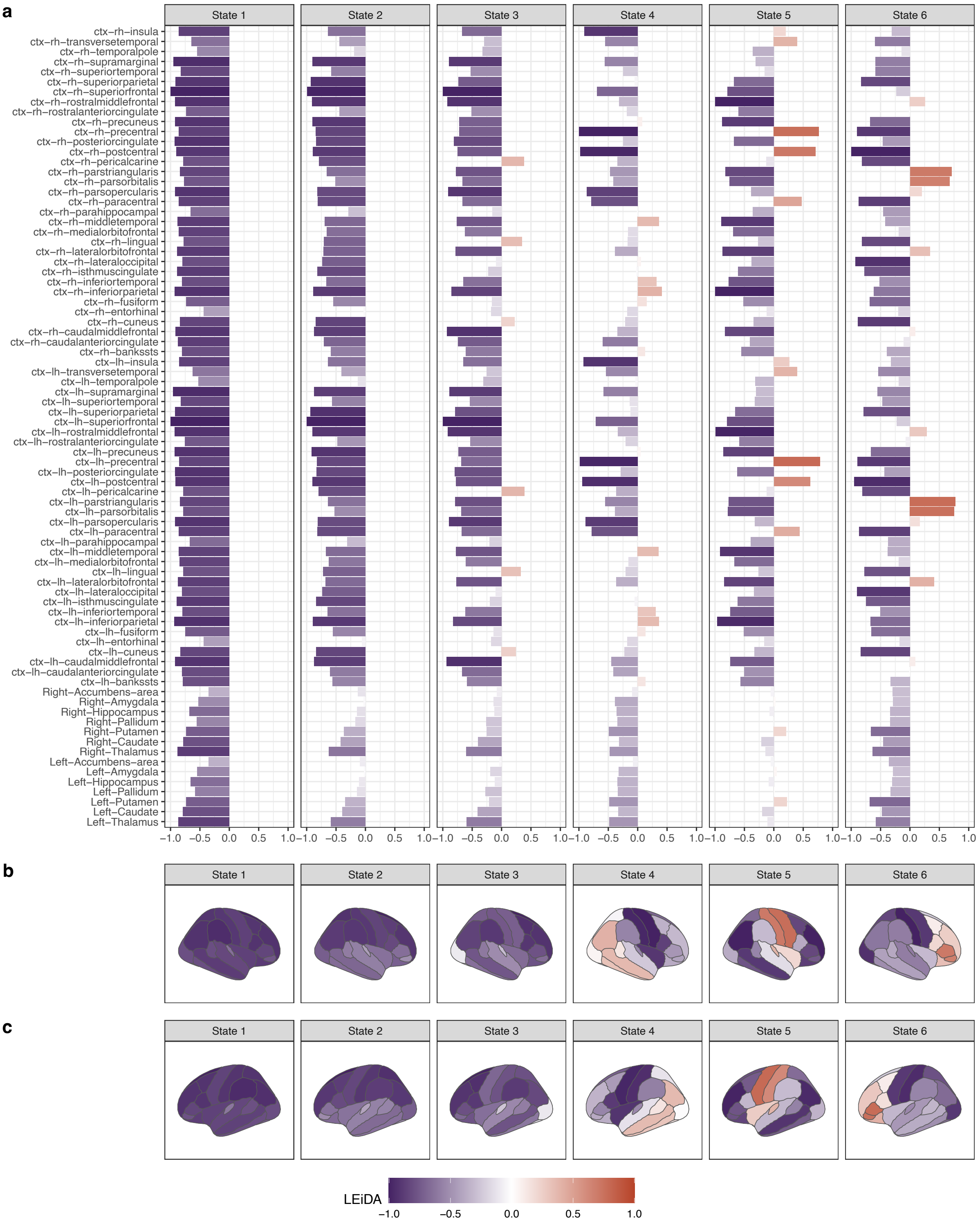

Supplementary Figure S3 | Brain states in neonates using M-CRIB atlas. (**a**) LEiDA vectors for each of the six brain states identified in the neonatal brain using M-CRIB parcels. (**b**) Representation of LEiDA on brain surfaces (right side view). (**c**) Representation of LEiDA on brain surfaces (left side view).

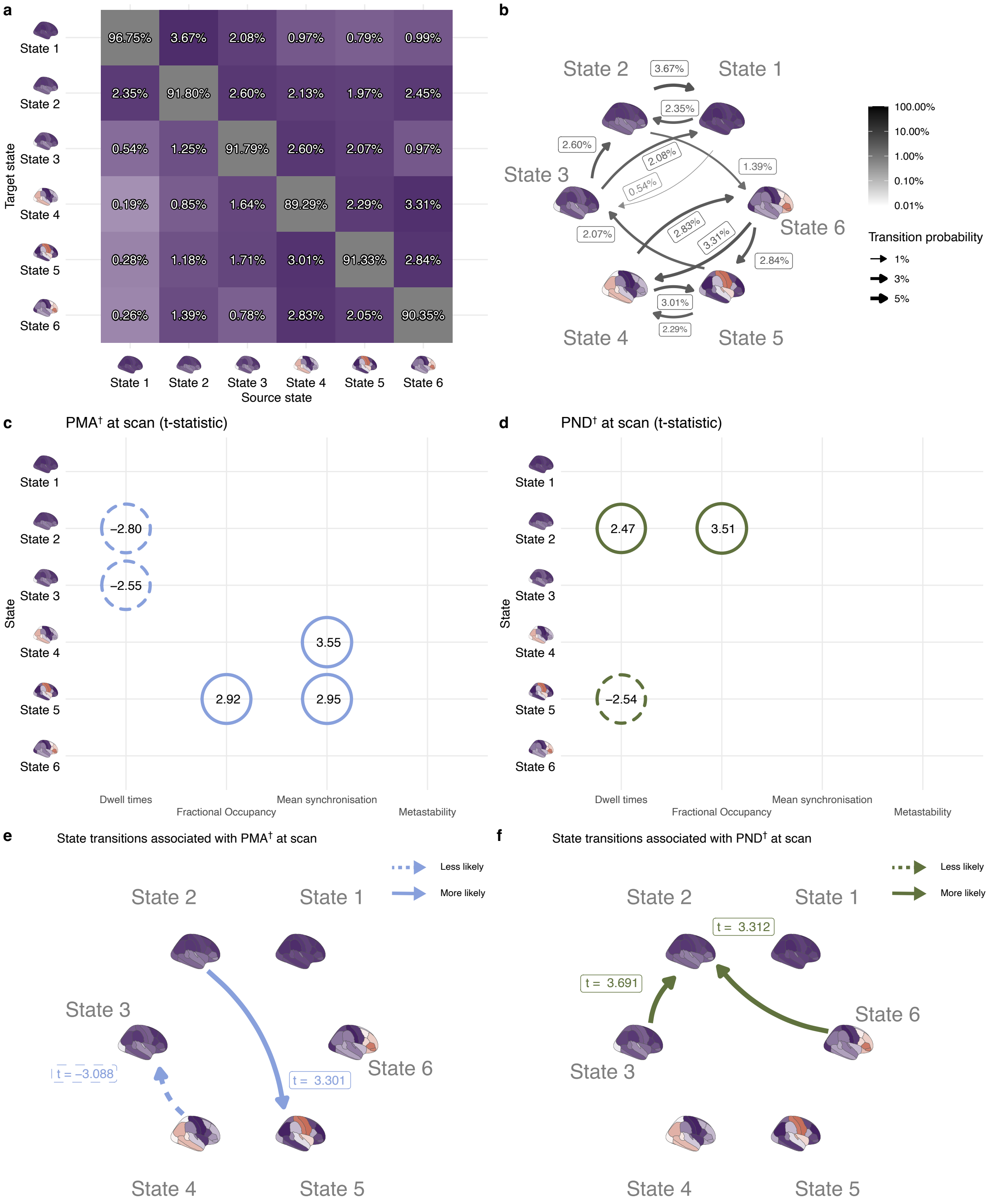

Supplementary Figure S4 | Brain dynamics in term-born neonates (n = 324) (M-CRIB atlas). (**a**) All transitions including dwelling state transitions. (**b**) Main transitions (top 12) between states excluding dwelling sequences. (**c**) Summary of brain state features significantly associated with PMA at scan. (**d**) Summary of brain state features significantly associated with PND at scan. (**e**) Summary of significant correlations between state transitions probabilities and PMA at scan. (**f**) Summary of significant correlations between state transitions probabilities and PND at scan. †GLM1 (including 324 term-born babies): $y \sim \beta_0 + \beta_1\text{PMA} + \beta_2\text{PND} + \beta_3\text{Sex} + \beta_4\text{Motion outliers}$ (FD). Values shown in **c** and **d** indicate t-statistics. All significant associations (two-sided permutation test) shown in this figure survive FDR multiple comparison correction with α error at 5%. PMA: Postmenstrual age. PND: Postnatal days.

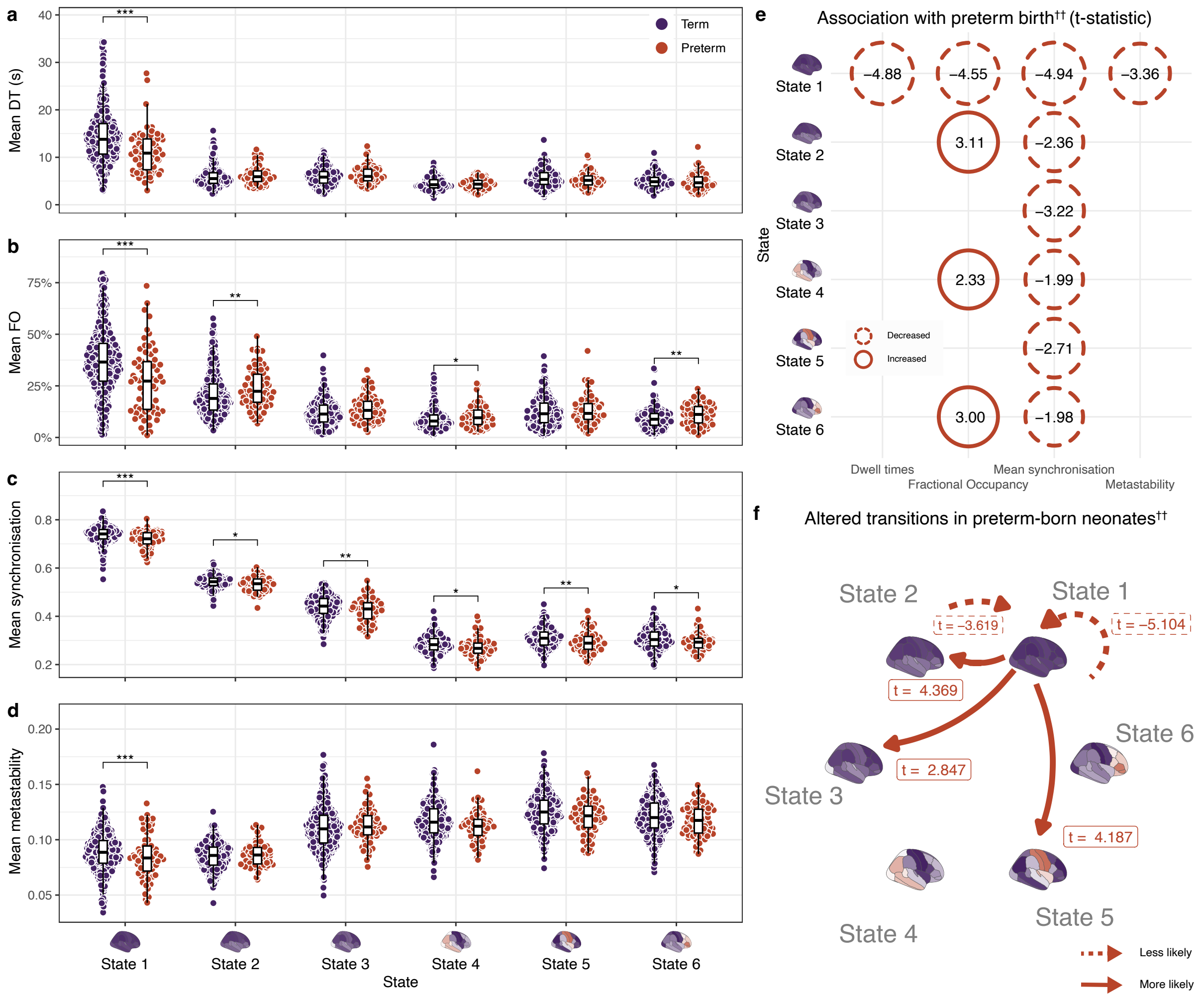

Supplementary Figure S5 | Brain dynamics in preterm-born neonates (M-CRIB atlas). (**a**) Mean dwell times (DT). (**b**) Mean fractional occupancy (FO). (**c**) Mean synchronisation. (**d**) Metastability. (**e**) Summary of significant associations with preterm birth (**f**) Association of state transitions probabilities and preterm birth. †GLM1 (324 term-born babies): $y \sim \beta_0 + \beta_1 PMA + \beta_2 PND + \beta_3 Sex + \beta_4 Motion\ outliers\ (FD)$. ††GLM2 (324 term-born and 66 preterm-born babies): $y \sim \beta_0 + \beta_1 Preterm\text{-}born + \beta_2 PMA + \beta_3 Sex + \beta_4 Motion\ outliers\ (FD)$. * $p < 0.05$. ** $p < 0.01$. *** $p < 0.001$ obtained with a two-sided permutation test. Values shown in **e** indicate t-statistics. Boxplots showing 0th, 25th, 50th, 75th and 100th centiles. Outliers defined when value larger than 1.5*IQR + 75th centile. All significant associations highlighted survive FDR multiple comparison correction with α error at 5%. PMA: Postmenstrual age. PND: Postnatal days. DT: Dwell time. FO: Fractional occupancy.

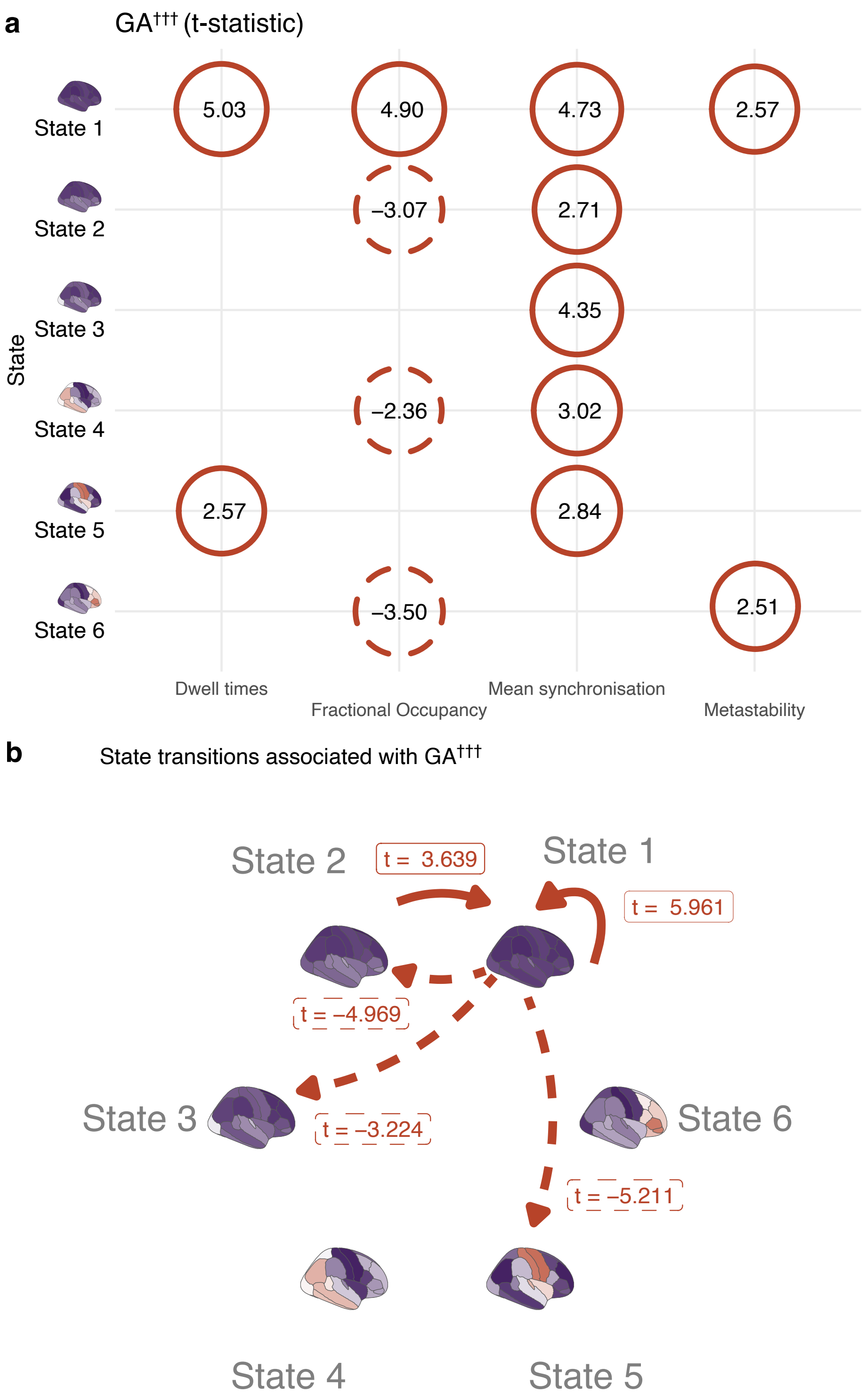

Supplementary Figure S6 | Association of GA at birth with modular features of neonatal brain dynamics (M-CRIB atlas). (**A**) Summary of associations between each of the four metrics (dwell times, fractional occupancy, mean synchronisation, metastability) and GA at birth. (**B**) Association of state transitions probabilities and GA at birth. †††GLM3 (including 324 term-born and 66 preterm-born babies): $y \sim \beta_0 + \beta_1 GA + \beta_2 PMA + \beta_3 Sex + \beta_4 Motion$ outliers (FD). * p < 0.05. ** p < 0.01. *** p < 0.001. Occ.: Occipital. SM: Sensorimotor. FP: Frontoparietal. PMA: Postmenstrual age. GA: Gestational age.

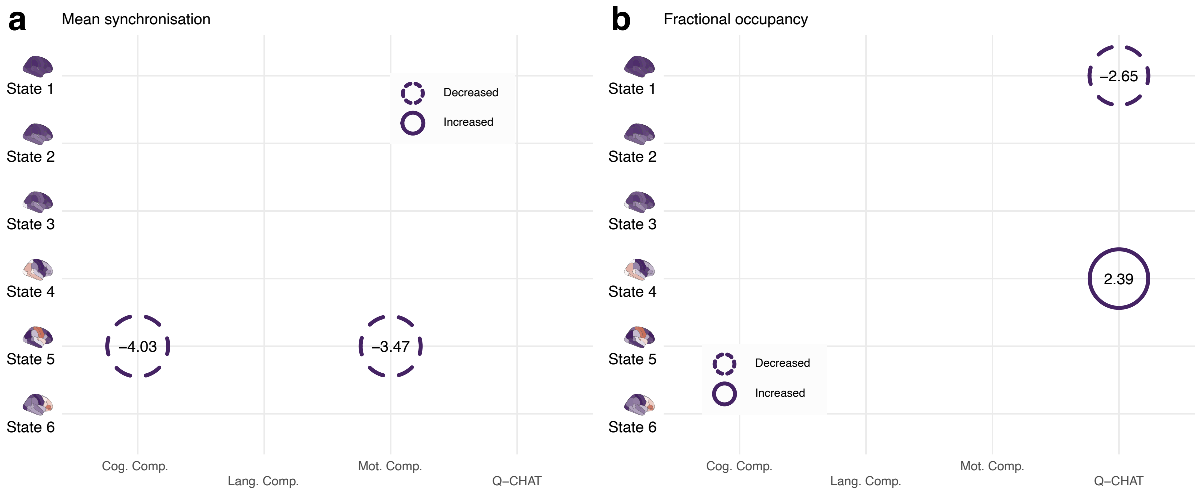

Supplementary Figure S7 | Summary of associations of brain state features with neurodevelopmental outcomes at 18 months corrected age (M-CRIB atlas). Association of average (**a**) mean synchronisation and (**b**) fractional occupancy in each of the six defined brain states during perinatal period with cognitive (Cog. Comp), language (Lang. Comp.) and motor (Mot. Comp) Bayley-III composite scores and Q-CHAT scores. GLM3 (257 term-born and 48 preterm-born babies for Bayley-III; and 254 term-born and 46 preterm-born babies for Q-CHAT): $y \sim \beta_0 + \beta_1$GA + β_2PMA + β_3Sex + β_4Motion outliers (FD) + β_5[Corrected age at assessment] + β_6[Assessed component] + β_7[Index of multiple deprivation]. Values shown in in both panels indicate t-statistics. All significant associations (two-sided permutation test) highlighted survive FDR multiple comparison correction with α error at 5%. PMA: Postmenstrual age. GA: Gestational age. Cog.: Cognitive. Lang.: Language. Mot.: Motor. Comp.: Component. Q-CHAT: Quantitative Checklist for Autism in Toddlers.

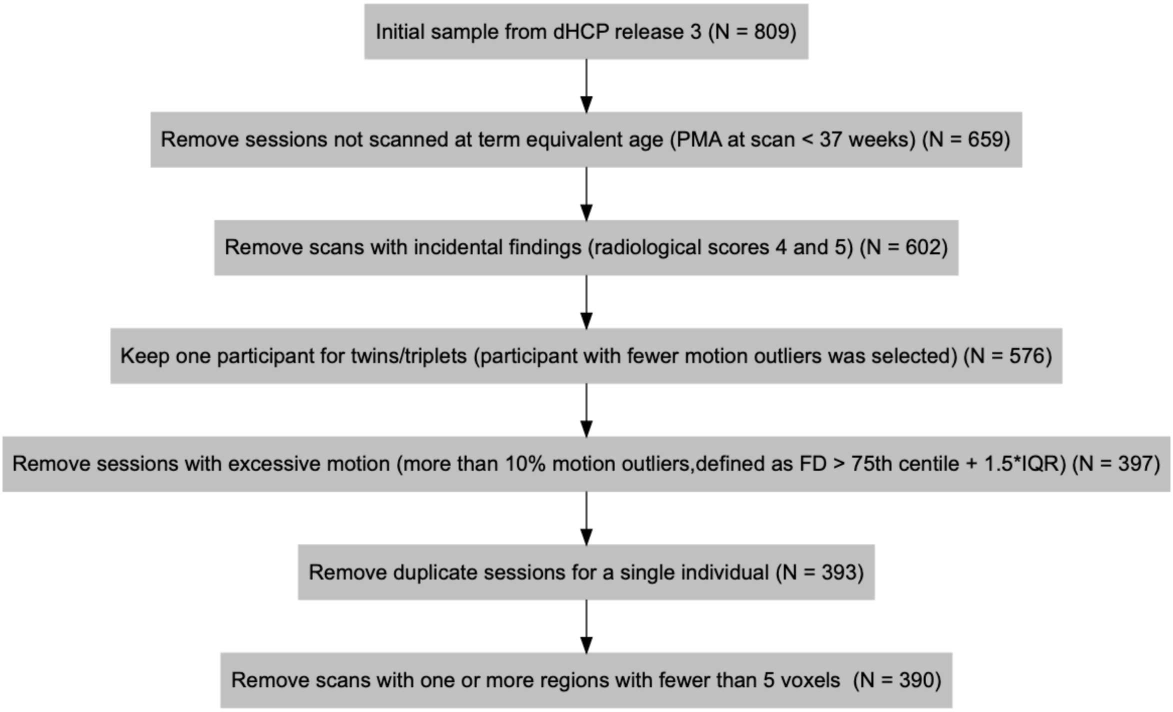

Supplementary Figure S8. Exclusion criteria flowchart.

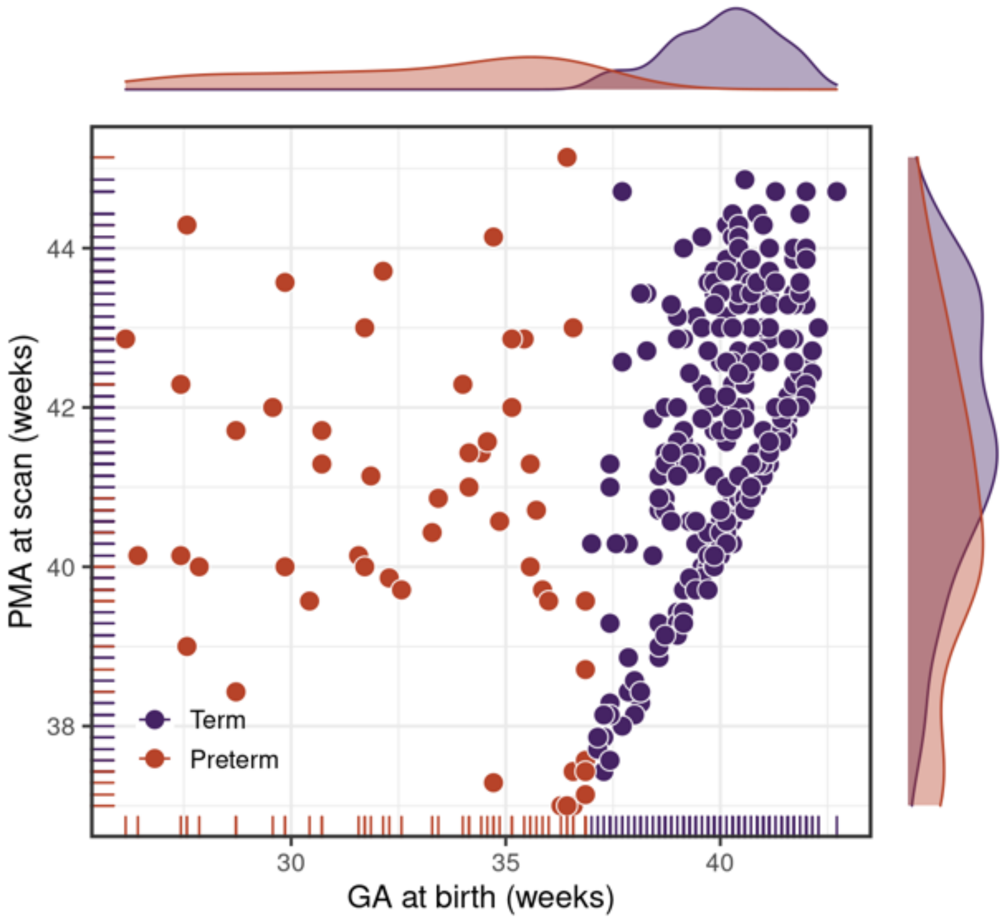

Supplementary Figure S9. Distribution of gestational age (GA) at birth and postmenstrual age (PMA) at scan for the participants included in this study.

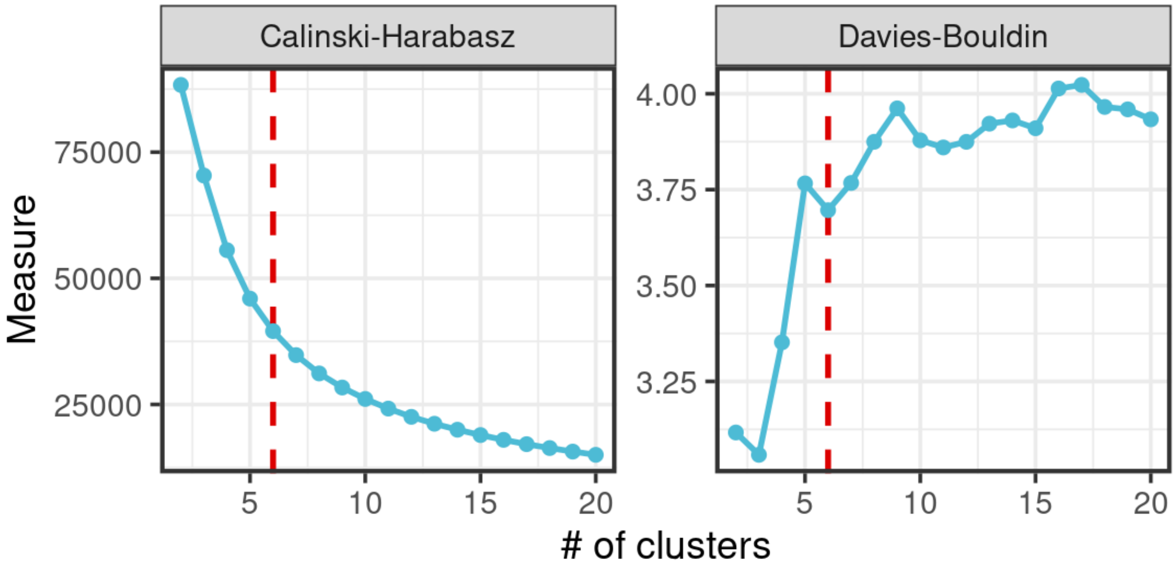

Supplementary Figure S10. Ideal number of clusters according to Calinski-Harabasz and Davies-Bouldin methods.

Supplementary Results: Association of neonatal brain regional brain dynamics with PMA at scan and GA at birth using an alternative brain parcellation (M-CRIB atlas)

The six brain states obtained with the alternative M-CRIB neonatal atlas were compatible with the ones obtained for the AAL atlas (Supplementary Figure S3), with three global synchronisation states and three more regionally constrained. States 1, 2 and 3 obtained with the M-CRIB atlas were consistent with global synchronisation states obtained with the AAL atlas. The M-CRIB's State 4 featured some of the structures present in Occipital state, State 5 featured similar structures to the sensorimotor state, and State 6 was concordant with FP state obtained from AAL.

In term-born babies, PMA at scan is associated with increased mean synchronisation in State 4 ($t = 3.6$; $p < 0.001$) and State 5 ($t = 2.9$; $p = 0.003$); and fractional occupancy in State 5 ($t = 2.9$; $p = 0.002$). PMA is also associated with reduced dwell times for States 2 ($t = -2.8$; $p = 0.004$) and 3 ($t = -2.6$; $p = 0.012$). Transitions from State 2 to State 5 increased ($t = 3.3$; $p = 0.001$) and transitions from State 4 to State 3 decreased ($t = -3.1$; $p = 0.003$) with PMA. Also, in term-born babies, PND is associated with increases in dwell times ($t = 2.5$; $p = 0.012$) and fractional occupancy ($t = 3.5$; $p < 0.001$; and reduced dwell times for State 5 ($t = -2.5$; $p = 0.012$). Transitions from States 3 and 6 to State 2 also increased with PND ($t = 3.7$; $p < 0.001$ and $t = 3.3$; $p = 0.002$, respectively). SM and State 5 exhibit compatible associations with PMA and PND (Supplementary Figure S4).

Preterm-birth is associated with reduced dwell times for State 1 ($t = -4.9$; $p < 0.001$); reduced fractional occupancy for State 1 ($t = -4.5$; $p < 0.001$); reduced mean synchronisation for the States 1 ($t = -4.9$; $p < 0.001$), 2 ($t = -2.4$; $p = 0.018$), 3 ($t = -3.2$; $p = 0.002$), 4 ($t = -2.0$; $p = 0.047$), 5 ($t = -2.7$; $p = 0.007$), and 6 ($t = -2.0$; $p < 0.05$); and reduced metastability for Stage 1 ($t = -3.0$; $p = 0.003$). We have also found an association with increased fractional occupancy for States 2 ($t = 3.1$; $p = 0.002$), 4 ($t = 2.3$; $p = 0.019$), and 6 ($t = 3.0$; $p = 0.003$). Transitions from State 2 to State 1 ($t = -3.6$; $p < 0.001$) and dwelling sequences of State 1 ($t = -5.1$; $p < 0.001$) are reduced in preterm birth. Transitions from State 1 to State 2 ($t = 4.4$; $p < 0.001$); State 1 to State 3 ($t = 2.8$; $p = 0.005$); and State 1 to State 5 ($t = 4.2$; $p < 0.001$) increased with preterm birth (Supplementary Figure S5 and Supplementary Figure S6).

Developmental outcomes in cognitive ($t = -4.0$; $p < 0.001$) and motor ($t = -3.5$; $p < 0.001$) components of Bayley-III show negative associations with mean synchronisation for State 5. Moreover, higher Q-CHAT scores were associated with reduced fractional occupancy for State 1 ($t = -2.7$; $p = 0.008$) and increased fractional occupancy ($t = 2.4$; $p = 0.017$), see Supplementary Figure S7 for a summary. The results for Mean synchronisation for State 5 are compatible with those shown in AAL atlas (SM state), as well as fractional occupancy and Q-CHAT for State 1.
