# Supplementary material for: Neonatal brain dynamic functional connectivity in term and preterm infants and its association with early childhood neurodevelopment
Source: Nat Commun. 2024 Feb 8;15:16. doi: 10.1038/s41467-023-44050-z (PMC10853532; doi:10.1038/s41467-023-44050-z)
Supplement: Supplementary file 3 — Reporting Summary [file 41467_2023_44050_MOESM3_ESM.pdf]

nature portfolio

Corresponding author(s): Dafnis Batalle

Last updated by author(s): Oct 6, 2023

Reporting Summary

Nature Portfolio wishes to improve the reproducibility of the work that we publish. This form provides structure for consistency and transparency in reporting. For further information on Nature Portfolio policies, see our Editorial Policies and the Editorial Policy Checklist.

Statistics

For all statistical analyses, confirm that the following items are present in the figure legend, table legend, main text, or Methods section.

n/a	Confirmed	
☐	☒	The exact sample size (*n*) for each experimental group/condition, given as a discrete number and unit of measurement
☐	☒	A statement on whether measurements were taken from distinct samples or whether the same sample was measured repeatedly
☐	☒	The statistical test(s) used AND whether they are one- or two-sided<br>*Only common tests should be described solely by name; describe more complex techniques in the Methods section.*
☐	☒	A description of all covariates tested
☐	☒	A description of any assumptions or corrections, such as tests of normality and adjustment for multiple comparisons
☐	☒	A full description of the statistical parameters including central tendency (e.g. means) or other basic estimates (e.g. regression coefficient) AND variation (e.g. standard deviation) or associated estimates of uncertainty (e.g. confidence intervals)
☐	☒	For null hypothesis testing, the test statistic (e.g. *F*, *t*, *r*) with confidence intervals, effect sizes, degrees of freedom and *P* value noted<br>*Give P values as exact values whenever suitable.*
☒	☐	For Bayesian analysis, information on the choice of priors and Markov chain Monte Carlo settings
☒	☐	For hierarchical and complex designs, identification of the appropriate level for tests and full reporting of outcomes
☐	☒	Estimates of effect sizes (e.g. Cohen's *d*, Pearson's *r*), indicating how they were calculated

Our web collection on statistics for biologists contains articles on many of the points above.

Software and code

Policy information about availability of computer code

Data collection	We used data collected as part of the Developing Human Connectome Project (http://www.developingconnectome.org). No custom algorithms were used for data collection as part of this manuscript.
Data analysis	We analyse fMRI data with dynFC: CoDe-Neuro's Dynamic Functional Connectivity Tools, a set of scripts written in Python v3.7 which we have made publicly available: https://code- neuro.github.io/dynfc/. A full set of script to allow the reproduction of the results presented in this manuscript is available in https://github.com/CoDe-Neuro/neonatal_dfc

For manuscripts utilizing custom algorithms or software that are central to the research but not yet described in published literature, software must be made available to editors and reviewers. We strongly encourage code deposition in a community repository (e.g. GitHub). See the Nature Portfolio guidelines for submitting code & software for further information.

Data

Policy information about availability of data

All manuscripts must include a data availability statement. This statement should provide the following information, where applicable:

- Accession codes, unique identifiers, or web links for publicly available datasets
- A description of any restrictions on data availability
- For clinical datasets or third party data, please ensure that the statement adheres to our policy

The fMRI datasets and clinical data analysed in this study are available as part of the dHCP's third data release, which can be obtained from https://data.developingconnectome.org. Pre-processed BOLD timeseries data used in this study, and adaptation of the AAL-UNC neonatal atlas to the dHCP template space

are available in https://dx.doi.org/ 10.5281/zenodo.7053984.

Human research participants

Policy information about studies involving human research participants and Sex and Gender in Research.

Reporting on sex and gender	Findings in this study only apply to sex.
Population characteristics	Our population consists of babies born at term and preterm and scanned between 38 and 42 weeks postmenstrual age.
Recruitment	Participants were recruited at Evelina London Children's Hospital from the local community. We are not aware of any potential self-selection bias that could be present and impact the results of this study.
Ethics oversight	Ethical approval was given by the UK National Research Ethics Authority (14/LO/1169).

Note that full information on the approval of the study protocol must also be provided in the manuscript.

Field-specific reporting

Please select the one below that is the best fit for your research. If you are not sure, read the appropriate sections before making your selection.

[x] Life sciences [ ] Behavioural & social sciences [ ] Ecological, evolutionary & environmental sciences

For a reference copy of the document with all sections, see nature.com/documents/nr-reporting-summary-flat.pdf

Life sciences study design

All studies must disclose on these points even when the disclosure is negative.

Sample size	We used all the data publicly available as part of the dHCP data release 3. Given the exploratory nature of this cohort study, we did not perform a sample size calculation. The final sample size was reached after applying our exclusion criteria to the data available in dHCP data release 3.
Data exclusions	fMRI datasets with excessive motion (more than 10% of motion outliers), or incidental MRI findings of clinical significance (major lesions within white matter, cortex, basal ganglia or cerebellum) were excluded. In cases of twin/triplet scans only one infant was included (the one with least motion outliers during acquisition). Number of participants excluded in each step are detailed in Supplementary Figure S3.
Replication	All datasets and scripts to allow the reproduction of our results are publicly available. We didn't attempt a replication in an independent sample.
Randomization	We used data from a cohort study with publicly available data (http://www.developingconnectome.org), hence randomisation was not applicable.
Blinding	We used data from a cohort study with publicly available data (http://www.developingconnectome.org), hence blinding the data was not applicable.

Reporting for specific materials, systems and methods

We require information from authors about some types of materials, experimental systems and methods used in many studies. Here, indicate whether each material, system or method listed is relevant to your study. If you are not sure if a list item applies to your research, read the appropriate section before selecting a response.

Materials & experimental systems

n/a	Involved in the study	
x		Antibodies
x		Eukaryotic cell lines
x		Palaeontology and archaeology
x		Animals and other organisms
	x	Clinical data
x		Dual use research of concern

Methods

n/a	Involved in the study	
x		ChIP-seq
x		Flow cytometry
	x	MRI-based neuroimaging

Clinical data

Policy information about clinical studies

All manuscripts should comply with the ICMJE guidelines for publication of clinical research and a completed CONSORT checklist must be included with all submissions.

Clinical trial registration	NA
Study protocol	NA
Data collection	Evelina Newborn Imaging Centre, Evelina London Children's Hospital. Recruited between 2014 and 2018.
Outcomes	NA

Magnetic resonance imaging

Experimental design

Design type	Resting state
Design specifications	We acquired one session/block per participant with a total duration of 15 minutes 3 seconds
Behavioral performance measures	NA

Acquisition

Imaging type(s)	fMRI + T2-weighted
Field strength	3 Tesla
Sequence & imaging parameters	Repetition time (TR) = 392 ms, echo time (TE) = 38 ms, voxel size = 2.15 x 2.15 x 2.15 mm, flip angle = 34, 45 slices, total time = 15 m 3 s, number of volumes = 2300
Area of acquisition	Whole brain

Diffusion MRI ☐ Used ☒ Not used

Preprocessing

Preprocessing software	dHCP neonatal resting-state functional preprocessing pipeline: https://doi.org/10.1016/j.neuroimage.2020.117303
Normalization	Data was analysed in native space
Normalization template	We used an AAL atlas mapped to the dhcp neonatal template which was then transformed into each subject's native space. To do so we used a non-linear registration based on a diffeomorphic symmetric image normalisation method (using ANTS software) with T2-weighted contrast and tissue segmentation as input channels.
Noise and artifact removal	As described in the dHCP neonatal processing pipeline: https://doi.org/10.1016/j.neuroimage.2020.117303
Volume censoring	We didn't censor volumes, but exclude subjects with more than 10% outliers, and used number of outliers as a covariate for statistical analyses.

Statistical modeling & inference

Model type and settings	General Linear Models (GLM) with relevant covariates to characterise main effects (age at scan, term/preterm, neurodevelopmental outcome). We evaluated the statistical significance of each variable of interest with random permutation tests with 10,000 repetitions for all GLMs.
Effect(s) tested	Resting-state, no task or stimulus condition tested.

Specify type of analysis: ☐ Whole brain ☒ ROI-based ☐ Both

Anatomical location(s)	AAL atlas adapted to the neonatal brain.
Statistic type for inference (See Eklund et al. 2016)	Statistics performed on metrics characterising dynamic brain states from temporal connectivity matrices
Correction	P-values are reported uncorrected, highlighting those surviving multiple comparison correction using the False Discovery Rate (FDR) method with alpha error at 5%

Models & analysis

n/a	Involved in the study
☐	☒ Functional and/or effective connectivity
☐	☒ Graph analysis
☐	☒ Multivariate modeling or predictive analysis

Functional and/or effective connectivity	Phase synchronisation
Graph analysis	Temporally resolved weighted graphs based on cosine distance of phases. No graph theory metrics were applied. Instead transient connectivity states were characterised.
Multivariate modeling and predictive analysis	LEiDA, K-Means clustering, Markovian modelling
